# Kirenol: A Potential Natural Lead Molecule for a New Drug Design, Development, and Therapy for Inflammation

**DOI:** 10.3390/molecules27030734

**Published:** 2022-01-23

**Authors:** Naurah Nabihah Nasir, Mahendran Sekar, Shivkanya Fuloria, Siew Hua Gan, Nur Najihah Izzati Mat Rani, Subban Ravi, M. Yasmin Begum, Kumarappan Chidambaram, Kathiresan V. Sathasivam, Srikanth Jeyabalan, Arulmozhi Dhiravidamani, Lakshmi Thangavelu, Pei Teng Lum, Vetriselvan Subramaniyan, Yuan Seng Wu, Abul Kalam Azad, Neeraj Kumar Fuloria

**Affiliations:** 1 Department of Pharmaceutical Chemistry, Faculty of Pharmacy and Health Sciences, Royal College of Medicine Perak, University Kuala Lumpur, Ipoh 30450, Perak, Malaysia; naurahnabihah@gmail.com (N.N.N.); peiteng1013@gmail.com (P.T.L.); 2 Faculty of Pharmacy, AIMST University, Bedong 08100, Kedah, Malaysia; azad@aimst.edu.my; 3 School of Pharmacy, Monash University Malaysia, Bandar Sunway 47500, Selangor, Malaysia; gan.siewhua@monash.edu; 4 Faculty of Pharmacy and Health Sciences, Royal College of Medicine Perak, University Kuala Lumpur, Ipoh 30450, Perak, Malaysia; najihah.izzti@gmail.com; 5 Department of Chemistry, Karpagam Academy of Higher Education, Coimbatore 641021, Tamil Nadu, India; ravisubban@rediffmail.com; 6 Department of Pharmaceutics, College of Pharmacy, King Khalid University, Abha 61421, Saudi Arabia; ybajen@kku.edu.sa; 7 Department of Pharmacology, College of Pharmacy, King Khalid University, Abha 62529, Saudi Arabia; kumarappan@kku.edu.sa; 8 Faculty of Applied Sciences, AIMST University, Bedong 08100, Kedah, Malaysia; skathir@aimst.edu.my; 9 Department of Pharmacology, Sri Ramachandra Faculty of Pharmacy, Sri Ramachandra Institute of Higher Education and Research (DU), Porur, Chennai 600116, Tamil Nadu, India; srikanth.j@sriramachandra.edu.in (S.J.); arulmozhidhiravidamani55@gmail.com (A.D.); 10 Center for Transdisciplinary Research, Department of Pharmacology, Saveetha Institute of Medical and Technical Sciences, Saveetha Dental College and Hospital, Saveetha University, Chennai 600077, Tamil Nadu, India; lakshmi@saveetha.com; 11 Faculty of Medicine, Bioscience and Nursing, MAHSA University, Jalan SP 2, Bandar Saujana Putra, Jenjarom 42610, Selangor, Malaysia; drvetriselvan@mahsa.edu.my; 12 Centre for Virus and Vaccine Research, School of Medical and Life Sciences, Sunway University, Subang Jaya 47500, Selangor, Malaysia; sengwu_21@yahoo.com; 13 Department of Biological Sciences, School of Medical and Life Sciences, Sunway University, Subang Jaya 47500, Selangor, Malaysia

**Keywords:** kirenol, inflammation, anti-inflammatory, molecular mechanism, drug development, molecular docking

## Abstract

Kirenol, a potential natural diterpenoid molecule, is mainly found in *Sigesbeckia* species. Kirenol has received a lot of interest in recent years due to its wide range of pharmacological actions. In particular, it has a significant ability to interact with a wide range of molecular targets associated with inflammation. In this review, we summarise the efficacy and safety of kirenol in reducing inflammation, as well as its potential mechanisms of action and opportunities in future drug development. Based on the preclinical studies reported earlier, kirenol has a good therapeutic potential against inflammation involved in multiple sclerosis, inflammatory bowel disorders, diabetic wounds, arthritis, cardiovascular disease, bone damage, and joint disorders. We also address the physicochemical and drug-like features of kirenol, as well as the structurally modified kirenol-derived molecules. The inhibition of pro-inflammatory cytokines, reduction in the nuclear factor kappa-B (NF-κB), attenuation of antioxidant enzymes, stimulation of heme-oxygenase-1 (HO-1) expression, and nuclear factor erythroid 2-related factor 2 (Nrf2) phosphorylation are among the molecular mechanisms contributing to kirenol’s anti-inflammatory actions. Furthermore, this review also highlights the challenges and opportunities to improve the drug delivery of kirenol for treating inflammation. According to the findings of this review, kirenol is an active molecule against inflammation in numerous preclinical models, indicating a path to using it for new drug discovery and development in the treatment of a wide range of inflammations.

## 1. Introduction

Pro-inflammatory cytokines such as the tumour necrosis factor-alpha (TNF-α), interleukin (IL)-1β, and vascular endothelial growth factor (VEGF) play important roles in inflammation [1]. Agents that inhibit the action of certain cytokines, or their receptors, restrict lymphocyte trafficking into tissues, prevent the binding of monocyte–lymphocyte co-stimulatory molecules, or deplete B lymphocytes have all been explored in the treatment of inflammation [1]. Regeneration and fibrosis are triggered by tissue injury and inflammation. Tissue injury not only causes inflammation, but also defines the type and polarisation of inflammation by recruiting and activating a number of innate and adaptive immune systems [2,3,4,5]. Additionally, multiple cell types and mediator signals are involved in the inflammatory response, which is a multi-stage process [6]. 

Inflammation is an adaptive response induced by a variety of signals, including microbial invasion or tissue damage [7]. Pathogen-associated molecular patterns (PAMPs) and damage-associated molecular patterns (DAMPs) are exogenous and endogenous “danger signals”, respectively [6,7,8]. Pattern recognition receptors (PRRs), such as Toll-like receptors (TLRs), recognise both PAMPs and DAMPs [9,10]. Pattern recognition receptor (PRR) activation triggers intracellular signalling pathways that include kinases and transcription factors [11]. For inflammation to develop, the signalling pathways listed above can promote the development of a range of inflammatory mediators, including cytokines. Acute and chronic inflammations are the two types of inflammation that can occur where the first is marked by pain, redness, and swelling, while the latter is marked by fatigue, fever, mouth sores, rashes, abdominal pain, and chest pain. Examples of inflammation include muscular dystrophy (muscle), multiple sclerosis (brain), asthma (lung), inflammatory bowel disorders (gastrointestinal tract), rheumatoid arthritis (bones), and atherosclerosis (heart) [12].

Anti-inflammatory medicines are divided into two categories: steroidal and non-steroidal anti-inflammatory drugs (NSAIDs). Most NSAIDs on the market today such as diclofenac, ibuprofen, naproxen, and a variety of others, have undesirable side effects. The NSAIDs target the cyclooxygenases (COX-1/2), which catalyse the first step from arachidonic acid to a variety of pro- and anti-inflammatory prostaglandins, leukotrienes, and thromboxanes, together with the lipoxygenases (LOX-5/12/15) [13]. Other important pathways include TLR-induced and mitogen-activated protein (MAP) kinase cascades, as well as glucocorticoid receptors that regulate transcription factors such as nuclear factor-activated T-cells (NFAT), nuclear factor-kappa B (NF-κB), and the signal transducer and activator of transcription 3 (STAT3). IL-1β, IL-6, IL-8, TNF-α, inducible nitric oxide synthase (iNOS), and COX-2 are among the pro-inflammatory mediators regulated and induced by the latter [14].

Many therapeutic methods are being tested in clinical trials due to the increased incidence of inflammatory diseases [15]. Nevertheless, the complexity and diversity of the human body′s inflammatory system pose a substantial difficulty in treating inflammatory diseases [12,16,17]. As a result, a thorough understanding of inflammatory processes is essential in the identification of new molecular targets and the development of new drugs to treat immune-related illnesses [18]. Furthermore, it is critical to discover novel anti-inflammatory medications with sufficient specificity, minimum side effects, and high efficacy from a variety of sources [18].

Many new anti-inflammatory medicines that suppress numerous inflammatory processes in the immune system are found in plants [19]. Researchers have spent decades searching for new anti-inflammatory drugs that are more effective, with fewer side effects, and are less expensive, particularly those derived from natural sources. The product may be chemically produced through either semi or total syntheses and is responsible for organic chemistry′s advancement by posing difficult synthetic targets. Natural products have good potential pharmacological properties that can be utilised to treat a wide range of illnesses. According to Li et al. [20], these compounds can be used as active components in both traditional and modern therapies. Natural compounds are frequently utilised as beginning points for drug discovery in chemical synthesis, serving as a foundation for the development of synthetic equivalents with a higher efficacy, potency, safety, and purity. 

**Figure 1:**
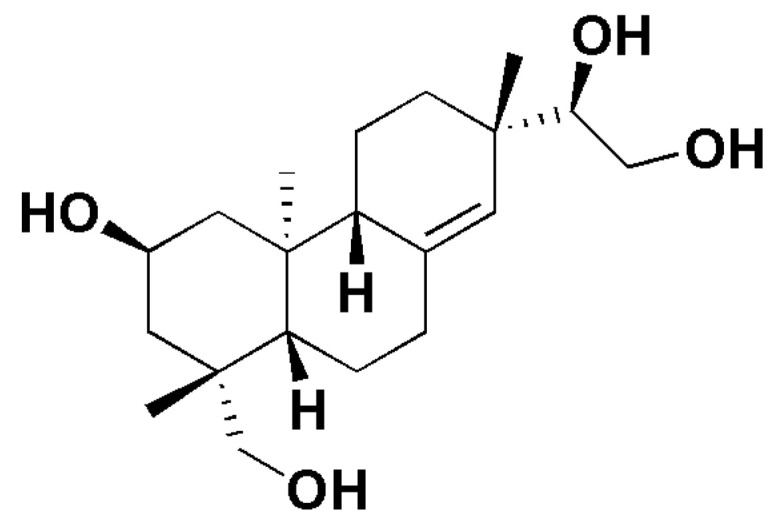
Chemical structure of kirenol.

Kirenol is a diterpenoid molecule (Figure 1) obtained primarily from *Sigesbeckia* species, which includes *Siegesbeckia orientalis*, *Siegesbeckia pubescens*, and *Siegesbeckia glabrescens* [21]. Kirenol has been associated with numerous biological effects, including anticancer, anti-inflammatory, cardioprotective, and antibacterial properties [21]. Although it has been reported for a variety of biological features, its anti-inflammatory potential is particularly promising for drug discovery and development. Hence, the current review focuses on the scientific evidence for kirenol′s anti-inflammatory potential as well as its possible mechanism of action. The physicochemical and drug-like features of kirenol are also addressed in this review, as well as the possibilities of structurally modified kirenol. In order to strengthen this review, we conducted molecular docking studies with selected proteins to corroborate its anti-inflammatory mechanism. The scientific evidence presented in this study is believed to provide a solid foundation for future research and vital information for the development of kirenol as an anti-inflammatory drug.

## 2. The Role of Kirenol against Inflammation

### 2.1. The Use of Kirenol against Complete Freund’s Adjuvant (CFA)-Induced Chronic Inflammation

**Figure 2:**
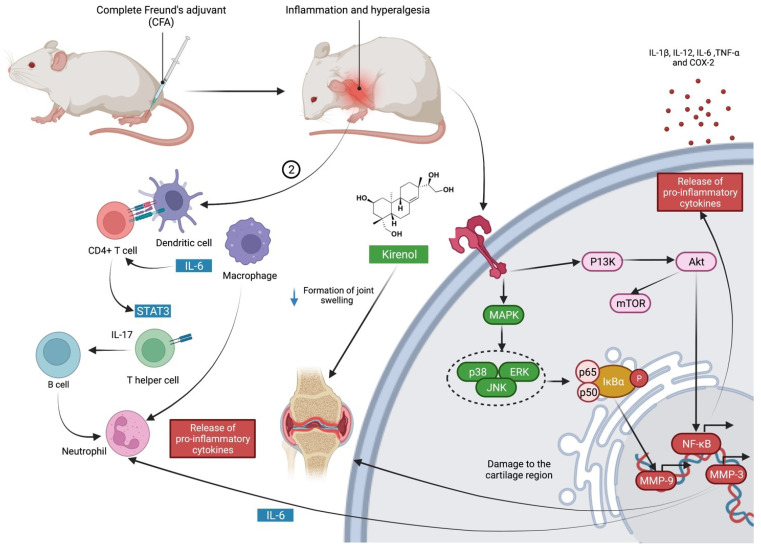
Possible mechanism of action of kirenol in the treatment of chronic inflammation generated by complete Freund′s adjuvant (CFA). CFA causes inflammation and hyperalgesia at the injection site due to the cellular and humoral antibody reactions to injected immunogens. The immune system of the sub-lining area (T cells, B cells, macrophages) is activated in response to CFA, which leads to the overproduction of pro-inflammatory mediators and antibodies. Reduced levels of TNF-α, MMP-3, IL-6, and IL-17, as well as increased release of pro-inflammatory mediators, can lead to increased joint inflammation. Kirenol modulates the immune response to minimise joint swelling caused by pro-inflammatory cytokines [24]. Abbreviations: STAT3, signal transducer and activator of transcription 3; IL-6, interleukin-6; IL-17, interleukin-17; IL-1β, interleukin-1 beta; IL-12, interleukin-12; TNF-α, tumour necrosis factor alpha; MAPK, mitogen-activated protein kinase; ERK, extracellular signal-regulated kinase; JNK, c-Jun N-terminal kinase; IkBa, nuclear factor of kappa light polypeptide gene enhancer in B-cells inhibitor alpha; P13K, phosphatidylinositol 3-kinase; mTOR, mammalian target of rapamycin; Akt, Ak strain transforming; MMP-9, matrix metallopeptidase-9; MMP-3, matrix metallopeptidase-3; NF-kB, nuclear factor kappa-light-chain-enhancer of activated B cells; cox-2, cyclooxygenase-2.

Complete Freund′s adjuvant (CFA) is a mineral oil-based adjuvant that contains a suspension of whole or pulverised heat-killed mycobacterium [22]. Its adjuvant effect is due to the continuous release of antigens from the oily deposit and promotion of a local innate immune response, resulting in a delayed hypersensitivity reaction with a severe inflammatory response and hyperalgesia at the injection site [23]. The administration of kirenol cream (0.3, 0.4 and 0.5% *w/w*) reduced the development of joint swelling caused by CFA, as evidenced by histopathological examinations [24]. Kirenol cream has been shown to have an anti-inflammatory effect comparable to piroxicam gel following carrageenan injection. The study concluded that this effect might be partially due to the production of pro-inflammatory cytokines such as IL-1β and TNF-α [24]. The possible mechanism of action of kirenol in the treatment of chronic inflammation caused by complete Freund′s adjuvant (CFA) is outlined in Figure 2 based on the overall results from the above findings [24].

### 2.2. The Use of Kirenol against Neuro-Inflammation

**Figure 3:**
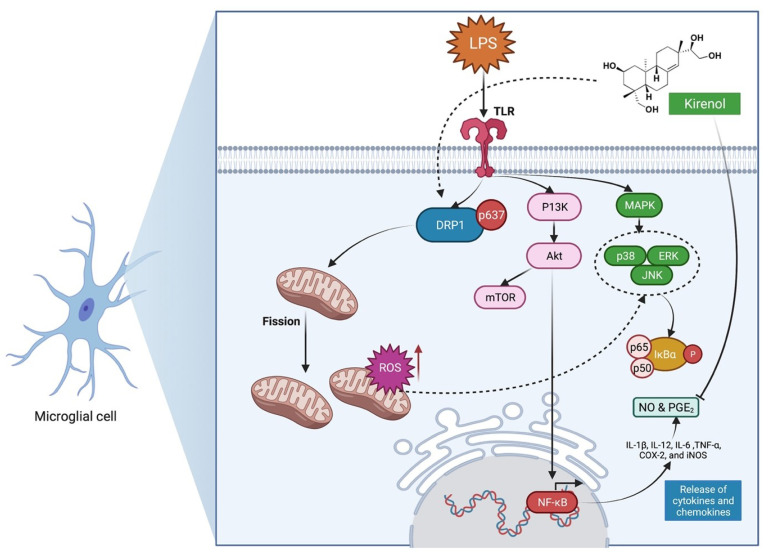
Possible mechanism of action of kirenol in the management of induced nitric oxide (NO) production in BV2 microglia by lipopolysaccharide (LPS). Inflammation-inducing elements such as LPS cause activated microglia to emit reactive oxygen species (ROS), cyclooxgenase-2 (COX-2), IL-1, IL-12, IL-6, iNOS, NO, and TNF-α. Kirenol inhibited microglia activation from triggering an inflammatory cascade that leads to the pathogenesis of neurodegenerative illnesses by lowering the levels of iNOS and COX-2 [27]. Moreover, kirenol also reduced Drp1 dephosphorylation attributed to ROS generation and pro-inflammatory response by suppressing excessive mitochondrial fission. Abbreviations: TLR, Toll-like receptor; DRP1, dynamin-related protein 1; IL-6, interleukin-6; IL-1β, interleukin-1 beta; IL-12, interleukin-12; TNF-α, tumour necrosis factor alpha; MAPK, mitogen-activated protein kinase; ERK, extracellular signal-regulated kinase; JNK, c-Jun N-terminal kinase; IkBa, nuclear factor of kappa light polypeptide gene enhancer in B-cells inhibitor, alpha; P13K, phosphatidylinositol 3-kinase; mTOR, mammalian target of rapamycin; Akt, Ak strain transforming; NF-kB, nuclear factor kappa-light-chain-enhancer of activated B cells; Cox-2, cyclooxygenase-2; iNOS, inducible nitric oxide synthase; ROS, reactive oxygen species, NO; nitric oxide; PGE_2_, prostaglandin E2.

The immune response of the central nervous system (CNS) (also known as “neuroinflammation”) is becoming more recognised as a key factor in the aetiology and progression of major neurodegenerative and mental illnesses [25]. In experimental autoimmune encephalomyelitis (EAE) mice, kirenol’s administration (10, 20 and 40 µM) significantly delayed disease onset and lowered clinical scores [26]. Kirenol’s administration lowered IFN-γ and IL-17A serum expressions, as well as the ratio of Th1 and Th17 cells in draining lymph nodes. In kirenol-treated EAE mice, lymphocyte priming was reduced and the apoptosis of myelin oligodendrocyte glycoprotein (MOG)-activated CD41T cells was increased. Based on the additional in vitro investigations by Xiao et al. [26], kirenol decreased the viability of MOG-specific lymphocytes and triggered apoptosis in MOG-specific CD41 T cells in dose- and time-dependent manners. Kirenol was observed to upregulate Bax expression, downregulate Bcl-2 expression, and increase caspase-3 activation and cytochrome c release, implying that kirenol-induced apoptosis involves a mitochondrial pathway [26]. This finding shows that kirenol may be a useful molecule in the treatment of multiple sclerosis. In addition to the results mentioned above, Lee et al. [27] found that kirenol (50 and 100 µM) inhibited lipopolysaccharide (LPS)-induced nitric oxide (NO) generation in BV2 microglia. Kirenol reduced the expression of iNOS and COX-2 in a concentration-dependent manner, as evidenced by quantitative real-time polymerase chain reaction (PCR) and Western blot analyses [27]. Microglia are crucial inflammatory cells in the CNS because the activation triggers an inflammatory cascade that contributes to the pathophysiology of neurodegenerative disorders. As a result of the abovementioned findings, kirenol appears to be a promising candidate for treating neuro-inflammation-related diseases, implying that kirenol′s anti-inflammatory properties may be useful in minimising the long-term effects of brain inflammation, such as cognitive deficiencies such as memory loss. Figure 3 depicts the possible mechanism of action of kirenol in the therapy of neuro-inflammation based on the above findings.

### 2.3. The Use of Kirenol against Cardiovascular Disease-Related Inflammation

Atherosclerosis is a chronic inflammatory illness characterised by artery dysfunction and, globally, is the primary cause of cardiovascular disease. According to Rajendran et al. [28], pretreatment with kirenol (5, 10, and 25 µM) significantly improved the cell survival of human umbilical vein endothelial cells (HUVECs), whereas DNA damage and the formation of reactive oxygen species (ROS) caused by benzo(a)pyrene (B(a)P) was inhibited. Kirenol′s potential as an antioxidant is directly associated with the increased expression of an antioxidant gene and the nuclear translocation activation of Nrf2, even in the absence of B(a)P, a ubiquitous environmental mutagen. Furthermore, this study established that Nrf2 translocation is mediated by the phosphatidylinositol 3-kinase (PI3K)/protein kinase B (AKT) signalling pathways as validated by the activation of repressed nuclear Nrf2 and the reduction in antioxidant genes in cells interacting with LY294002 [28]. Nevertheless, the protective effects of kirenol on HUVECs cells against oxidative damage are removed with knocked-down Nrf2 via Nrf2-siRNA transfection. Furthermore, kirenol′s ability to reduce endothelial dysfunction was corroborated by the production of apoptotic proteins. Additionally, kirenol appeared to counteract a B(a)P-induced redox imbalance in the vascular endothelium by activating Nrf2 signalling via the PI3K/AKT pathway [28]. Nevertheless, additional research is required to help in further understanding kirenol′s mechanism(s) of action against atherosclerosis, particularly in a clinical setting.

### 2.4. The Use of Kirenol against Lung Injury-Related Inflammatory Disease

**Figure 4:**
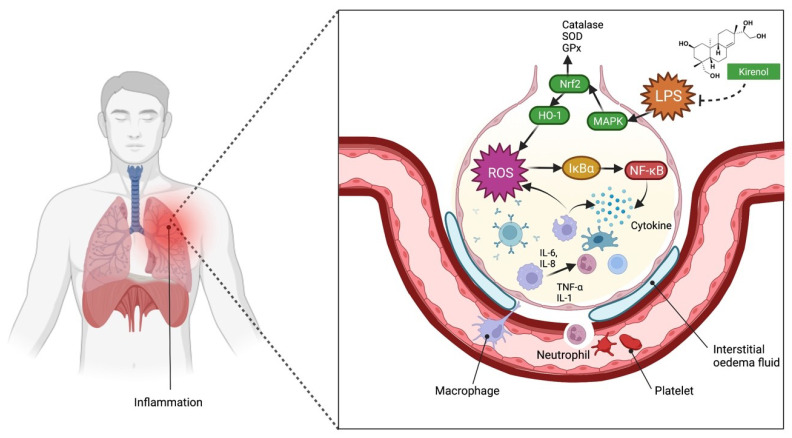
Possible mechanism of action of kirenol in the management of lung injury-related inflammatory disease. Several intracellular signalling pathways, including MAPK pathways, are stimulated by the LPS. LPS activates macrophages, resulting in increased production of inflammatory mediators and ROS. On LPS-treated macrophages, the key pro-inflammatory mediators TNF-α, interleukins, and nitric oxide (NO), which is produced by NO synthase, are upregulated, fostering the development of inflammatory disorders. HO-1 is released thought due to an adaptive cellular response to inflammation and oxidative damage, regulated by Nrf2 [29]. Kirenol reduces the histopathological alterations caused by lipopolysaccharide (LPS), such as interstitial oedema and leukocyte infiltration. Kirenol reduces the downregulation of the antioxidant enzymes as well as the release of cytokines TNF-α, IL-1, and IL-6. Abbreviations: IL-6, interleukin-6; IL-8, interleukin-8; IL-1, interleukin-1; Nrf2, nuclear factor-erythroid factor 2-related factor 2; MAPK, mitogen-activated protein kinase; IkBa, nuclear factor of kappa light polypeptide gene enhancer in B-cells inhibitor alpha; NF-kB, nuclear factor kappa-light-chain-enhancer of activated B cells; ROS, reactive oxygen species; HO-1, heme oxygenase-1.

Pretreatment with kirenol (30, 50 and 100 mg/kg, i.p.) reduced leukocyte infiltration in bronchoalveolar lavage fluid (BALF), disrupting the alveolar-capillary barrier and lipid peroxidation in lung tissues caused by LPS [29]. Through NF-κB activation, kirenol reduced the release of cytokines such as IL-1β, IL-6, and TNF-α into the BALF of mice with LPS-induced acute liver damage. Furthermore, kirenol inhibited the LPS-induced downregulation of antioxidant enzymes, including superoxide dismutase, glutathione peroxidase, and catalase (Figure 4). Kirenol stimulated the expression of HO-1 as well as the phosphorylation of Nrf2 and AMPK2. Kirenol can be developed as a therapy for acute lung injury by inhibiting the NF-κB pro-inflammatory pathway, promoting AMPK2/Nrf2-mediated HO-1, and antioxidant enzyme (AOE) activation [29]. Additionally, kirenol potentially ameliorates lung injury. Overall, despite the fact that the anti-inflammatory mechanism and underlying targets are unclear, kirenol′s favourable effects on LPS-induced inflammation make it an important lead molecule in future drug development and research. Figure 4 summarizes and demonstrates the possible mechanism of action of kirenol in the management of lung injury-related inflammatory disease.

### 2.5. The Use of Kirenol against Colon Injury

**Figure 5:**
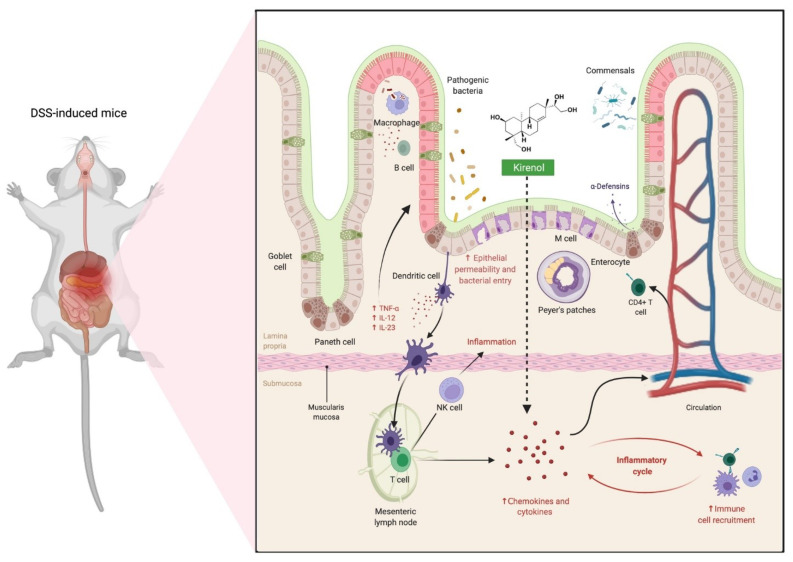
Possible mechanism of action of kirenol in the treatment of colon injuries. In a normal situation, macrophages are critical for maintaining local homeostasis, which is a balance of inflammatory and protective responses. When mice were given dextran sulphate sodium (DSS), experiencing colon damage or bacterial invasion, the number of macrophages rose dramatically, causing mucosal inflammation to begin with. Infiltrated bacteria engaged with macrophages, dendritic cells, and neutrophils, then further activated the innate immune receptors causing the production of proinflammatory cytokines and chemokines, accelerating the inflammatory reaction and inducing progression of irritable bowel syndrome (IBD). Kirenol reduces the release of IFN-γ, as well as other cytokines and chemokines, in the mesenteric lymph nodes (MLNs) [31]. Abbreviations: NK cell, natural killer cell; IL-6, interleukin-6; IL-17A, interleukin-17A; TNF-α, tumour necrosis factor alpha; IFN-γ, interferon gamma.

Irritable bowel disease (IBD) is a group of disorders characterised by a chronic and relapsing inflammatory disorder of the large intestine with the destruction of the mucosal barrier where ulcerative colitis is one of the most common types [30]. The administration of kirenol (2 mg/kg, p.o. for 7 days) reduced ulcerative colitis symptoms and the milder pathological abnormalities in the colon of dextran sodium sulphate (DSS)-induced colitis in mice [31]. Additionally, kirenol administration to DSS-induced mice reduced the release of IFN-γ, IL-17A, IL-6, and TNF-α by lymphocytes in the mesenteric lymph nodes (MLNs) and increased lymphocyte death, particularly in CD4^+^ T cells (Figure 5). Kirenol also protected DSS-induced mice against T cell-mediated colon damage by reducing the release of the inflammatory mediator and promoting apoptosis in inflammatory lymphocytes [31]. Taken together, the findings indicate that kirenol is a promising therapeutic compound to be used against T cell-driven colitis. Figure 5 depicts the possible mechanism of action of kirenol in the treatment of colon injuries.

### 2.6. The Use of Kirenol against Diabetic Wounds

Diabetes is a condition that is known to be linked to a variety of connective tissue abnormalities. Extended inflammation, an impaired development of new blood vessels, decreased collagen production, higher levels of protein enzymes, and dysfunctional macrophages are all symptoms of diabetic abrasion, resulting in delayed wound healing [32,33,34]. Ren et al. [35] investigated the anti-inflammatory role of kirenol in hyperglycaemic rats induced by streptozotocin. Kirenol treatment reduced the expression of NF-κB, COX-2, iNOS, MMP-2, and MMP-9. A further histological analysis revealed an improved shape of granule-forming tissue with a noticeable fibroblast propagation, amplified vascular initiation, and sedimentation of collagen fibres. Based on the above findings, Ren et al. [35] suggested that kirenol can ameliorate hyperglycaemic conditions and may be useful in the treatment and management of diabetic patients with recurrent lesions.

### 2.7. The Use of Kirenol against Bone Damage

Osteoporosis is a systemic bone disease that leads to the loss of bone mass, bone quality, and microarchitectural degeneration, resulting in a lower bone strength and a higher risk of fractures [36,37]. Kirenol significantly reduced osteoclast formation and bone resorption [38]. Additionally, it reduced the formation of the F-actin ring and suppressed the receptor activator of the NF-κB ligand (RANKL)-induced NF-κB p65 activation, as well as the expression of p-p38, p-ERK, and c-Fos. Furthermore, kirenol decreased the NFATc1 expression as well as nuclear translocation. In the in vitro model, kirenol reduced Ca^2+^ oscillation and caveolin-1 (Cav-1) during osteoclastogenesis. In vivo, kirenol (2–10 mg/kg) reduced ovariectomy (OVX)-induced osteoporosis, as demonstrated by the decrease in the osteoclast number and the downregulation of Cav-1 and NFATc1 expression. Additionally, kirenol protects against OVX-induced osteoporosis by suppressing osteoclastogenesis and the Cav-1/NFATc1 signalling pathways both in vitro and in vivo. The abovementioned study suggests that kirenol is a safe and effective oral therapy for osteoporosis [38]. In addition to the above study, the effects of kirenol (10, 20 and 40 µM) on osteoblast differentiation were accompanied by the increased expression of BMP and Wnt/β-catenin signalling pathways, including BMP2, runt-related transcription factor 2 (Runx2), osterix (Osx), low-density lipoprotein receptor related protein 5 (LRP5), dishevelled 2 (DVL2), β-catenin, cyclin D1, and phosphorylated glycogen synthase kinase 3β (GSK3β) [39]. Further, kirenol also up-regulated the expression of β-catenin, CCND1, ALP, and ColA1, which are down-regulated by β-catenin siRNA knockdown. Overall, the above findings indicate that kirenol can promote osteoblast development in MC3T3-E1 cells by activating the BMP and Wnt/β-catenin signalling pathways; thus, implying that it could be a suitable target for treating or preventing osteoporosis [39]. In addition, kirenol treatment (2 and 4 mg/kg, p.o. for 21 days) promoted fracture healing in a dose-dependent manner, with the early activation of the Wnt/β-catenin and activation of the Runx-2 pathways [40]. Overall, kirenol can be used as a natural lead molecule in the development of a drug for the prevention and treatment of bone deterioration such as osteoporosis.

### 2.8. The Use of Kirenol against Arthritis

Wu et al. [41] found that kirenol (100–200 µg/mL) reduced the migration, invasion, and pro-inflammatory IL-6 secretion of rheumatoid arthritis (RA)-associated synovial fibroblasts (FLS). In a collagen-induced arthritis (CIA) mice model, kirenol administration decreased pro-inflammatory cytokine production, synovium hyperplasia, and cartilage erosion. Due to the potential to reduce negative FLS activities, it is plausible that kirenol has potent therapeutic efficacy in RA [41]. Furthermore, kirenol (1, 2, and 4 mg/kg) decreased paw oedema and synovial fluid IL-1β in CIA rats. Kirenol reduced NF-κB activity in the CIA synovium by upregulating nuclear Annexin-1. Kirenol′s inhibitory effect on NF-κB activity was boosted by anti-Annexin-1 antibodies. Kirenol has a similar anti-inflammatory mechanism with glucocorticoids, albeit without the drawbacks of glucocorticoids treatment [42].

Furthermore, kirenol dramatically delayed the onset of collagen-induced arthritis (CIA) and lowered the severity of the condition [43]. A histological analysis confirmed that kirenol reduced joint inflammation and prevented cartilage and bone damage. Kirenol also enhanced the number of CD4^+^CD25^+^Foxp3^+^ and IL4^+^CD4^+^ T cells, while decreasing the frequency of IFN^+^CD4^+^ T cells by upregulating Foxp3 mRNA expression. TNF-α, IL-17A, and IL-6 levels in synovial fluid and TNF-α, IL-17A, and IFN-γ in serum were lowered by kirenol, whereas IL-4, IL-10, and TGF-β1 serum levels were elevated. In addition, compared to cells from CIA rats, kirenol decreased the ability of bovine type II collagen (CII) to stimulate splenocyte, peripheral blood mononuclear cells, and lymph node cell proliferation. These findings imply that kirenol can be used to treat rheumatoid arthritis by acting as an immunosuppressant [44]. Kirenol treatment (2 mg/kg, p.o. for 30 days) (1) reduced pro-inflammatory cytokine secretion (IFN-γ and TNF-α), (2) increased anti-inflammatory cytokine production (IL-10 and IL-4), (3) inhibited cell proliferation, and (4) induced the apoptosis of CII-specific lymphocytes, overall implying that kirenol may have immunosuppressive properties [44]. 

In another study, Wu et al. [45] employed an in silico tool (Auto Dock 4.2) to investigate the effect of kirenol on three major cytokine targets: TNF-α, IL-1β, and IL-6. All three cytokines showed spontaneous kirenol binding, with ΔG values ranging from −6.75 to −2.68 kcal/mole. Overall, the findings revealed that IL-6 is the greatest target (in terms of binding energy and the number of interactions) for kirenol when compared to TNF-α and IL-1β [45]. All of the above findings are important in furthering kirenol′s therapeutic potential in the treatment of autoimmune arthritis.

## 3. Overview of Mechanism of Action of Kirenol against Inflammation

TNF-α is a significant mediator for inflammation in most disorders, where its action is regulated by the activation of nuclear factor NF-κB, a transcription factor. Despite the fact that TNF-α is one of the most powerful NF-κB activators, NF-κB regulates TNF-α expression as well. NF-κB can be activated by inflammatory cytokines, Gram-negative bacteria, a variety of disease-causing viruses, environmental toxins, chemical, physical, mechanical, and psychological stress, excessive glucose, fatty acids, UV radiation, cigarette smoke, and other disease-causing factors [46,47,48,49,50]. Hence, medicines that inhibit NF-κB and NF-κB-regulated gene products may be useful in the treatment of a wide range of inflammatory diseases. Kirenol inhibits the production of COX which is typically induced by inflammation [27,35]. Excessive NO generation in activated microglia as induced by iNOS is linked to the advancement of neurodegenerative and inflammatory illnesses [51]. Kirenol inhibits the level of NO, TNF-*α,* and iNOS. It also increases the gene expression and production of Th-1-derived interferon-γ (IFN-γ), while suppressing Th-2-derived IL-4. Kirenol (1) downregulates the production of NF-κB, (2) stimulates anti-inflammatory cytokine IL-4 and IL-10, and (3) blocks IL-1β, IL-6, and IFN-γ. A growing body of research suggests that kirenol is an effective molecule in ameliorating inflammatory diseases, including allergic inflammations [24,26,27,28,29,35,38,39,40,41,42,43,44,45].

## 4. In Silico Molecular Docking Study

**Figure 6:**
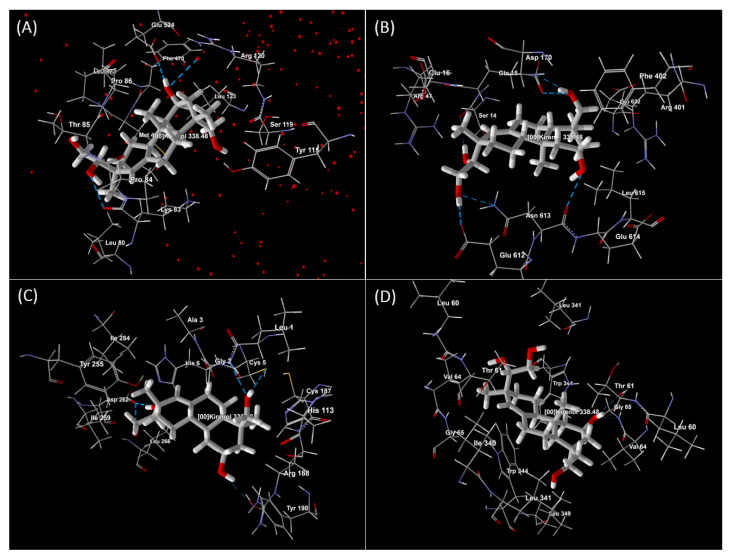
Docked view of kirenol on protein targets (**A**) COX-2 (PDB ID: 5IKR), (**B**) LOX-5 (PDB ID: 6NCF), (**C**) CXCR4 (PDB ID: 4RWS), and (**D**) human prostaglandin E receptor EP3 (PDB ID: 6AK3).

To support the anti-inflammatory potential and mechanism of action of kirenol, an in silico molecular docking study was performed with COX-2, lipoxygenase-5 (LOX-5), chemokine receptor 4 (CXCR4), and human prostaglandin E receptor EP3. COX-2 catalyses the oxygenation of arachidonic acid and endocannabinoid substrates, putting it at the junction of the eicosanoid and endocannabinoid signalling pathways for inflammation (PDB ID 5IKR) [52]. Leukotrienes (LT) are inflammatory lipid mediators that have been associated with asthma and atherosclerosis. LOX-5 initiates LT production at the nuclear membrane with the help of the substrate-binding LOX-5-activating protein (PDB ID: 6NCF) [53]. CXCR4 is a seven-transmembrane G protein-coupled receptor for chemokines. Chemokines and their receptors are involved in cell migration throughout development, immunological responses, and a variety of illnesses, including inflammation and cancer (PDB ID: 4RWS) [54,55]. Prostanoids are a class of bioactive lipid metabolites that act autacoid by activating cognate G-protein-coupled receptors, a process that plays a key role in inflammation (PDB ID: 6AK3) [56]. The protein structure of these targets was obtained from a protein data bank and prepared for a docking study by Molegro Virtual Docker 6.0. The docking results were examined using the Pose Organizer and the ligand energy inspector tool, and the results were tabulated and the docked view was extracted (Table 1 and Figure 6). It was revealed that the molecular docking score with the lowest values had the highest binding affinity to the target proteins. According to the report obtained from our study, the kirenol affinity for inflammatory targets was 5IKR > 6NCF > 4RWS > 6AK3. In silico approaches are becoming increasingly significant in drug research, and they are critical for quickly identifying viable therapeutic options. This finding supports and correlates well with kirenol′s anti-inflammatory potential reported in in vivo models. The researchers/scientists, on the other hand, do not believe the in silico results because they are generated by a computer. Thus, in vitro and in vivo investigations with experimental confirmation are required to ensure that in silico data are trustworthy and reliable. The outcomes of this study showed that the in silico model is reliable and could be used in the identification and development of anti-inflammatory drugs. The in silico findings from this study could accelerate the development of potential new medicines, particularly for the treatment of inflammation.

## 5. Challenges and Opportunities of Kirenol to Be Developed into a Drug Molecule for the Treatment of Inflammation

According to the data obtained from the DruLiTo software (Table 2), kirenol appears to be a promising drug-like molecule with the potential to be a good therapeutic agent for a range of disorders, including inflammation. To date, acute oral toxicity studies reported no mortality in rats administered with 2000 mg/kg of kirenol orally over a 14 days period, where there were no changes in the investigated parameters among the experimental rats during the monitoring period [24]. Using a toxicity estimation software tool such as the QSAR methodologies consensus method, hierarchical clustering method, and nearest neighbour method, the oral rat LD_50_ mg/kg (predicted value) of kirenol was found to be 6003.02 mg/kg, 5872.13 mg/kg, and 6136.82 mg/kg, respectively. Song et al. [57] successfully implemented a pharmacokinetic study of kirenol following oral treatment in rats. For the simultaneous determination of kirenol, an accurate and quick Liquid Chromatography Electrospray Ionization Tandem Mass Spectrometric (LC-ESI–MS/MS) technique was devised and validated [58]. In an in vivo model, kirenol appeared to be quickly absorbed and removed [59]. The in vivo instability, low bioavailability and solubility, poor absorption in the body, difficulty with target-specific delivery, tonic effectiveness, and possible adverse effects are all challenges occurring with the use of large-sized materials for drug delivery.

Inflammatory disorders must be treated using targets that are found in both healthy and diseased tissues. To achieve therapeutic effects on inflammatory cells, substantial medication doses are frequently required, which can cause undesired side effects in other tissues. As a result, while the present target-based drug design paradigm effectively identifies candidate medications that are molecularly selective, cell-type specificity is lacking, preventing the in vivo utility at efficacious levels. Hence, adopting innovative drug delivery methods to tailor the delivery of agents to specific body areas is a viable solution to pressing problems [60,61]. Therefore, nanotechnology is playing a key role in enhanced drug formulations, targeted arenas, and controlled medication release and delivery, where target-specific drug delivery methods such as dendrimers, micelles, and liposomes commonly incorporate metallic, organic, inorganic, and polymeric nanostructures.

Because of the high surface area to volume ratio of nanocarriers, the drug solubility and dissolution rate can be improved. Small particle sizes can also help medications stay in the systemic circulation for longer, increase drug distribution, and enabling drug targeting and a trans-barrier transfer. Further, nanocarriers have been employed to improve the physicochemical and pharmacokinetic properties of molecules. In many inflammatory illnesses such as arthritis, nano-emulsion and nanogel formulations can improve the penetration and accumulation of drug in the skin via local administration, which could be used as a future research direction of this molecule.

**Figure 7:**
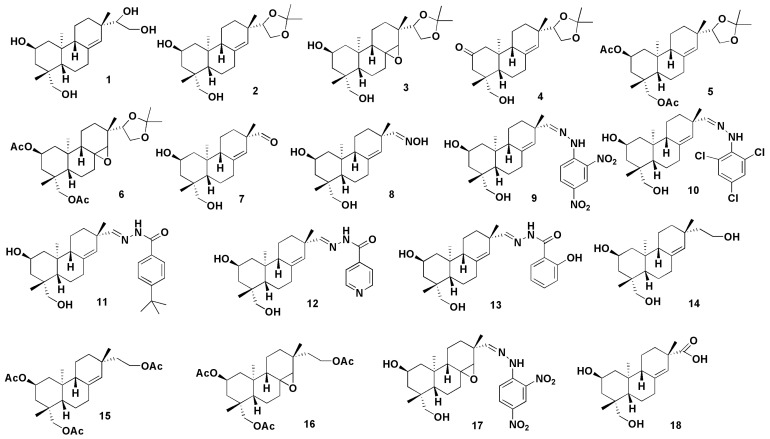

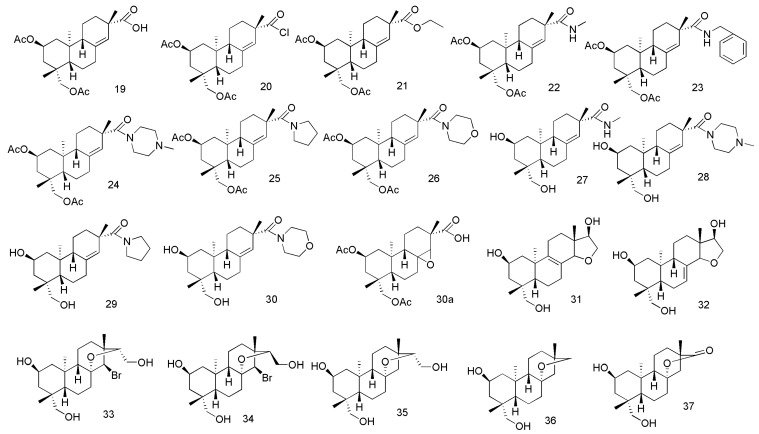
Possible structural modifications and derivatives of kirenol [62,63,64].

Additionally, structural modifications of kirenol may improve its physicochemical and pharmacokinetic properties. To investigate the pharmacological effects of kirenol, researchers changed the functional groups on its structure and generated a range of kirenol derivatives. Compounds **2–30a** were also developed to improve kirenol′s anti-inflammatory and cytotoxic properties [62]. Kirenol derivatives displayed limited cytotoxic action, according to the data. All of the investigated derivatives (**2–30a**) demonstrated considerable anti-inflammatory action, with seven of them (**5, 10, 15, 18, 25, 29 and 30a**) showing activities that exceeded that of the positive control (hydrocortisone, IC_50_ = 1.17 ± 0.8 μM) especially compound **18** (IC_50_ = 0.94 ± 0.1 μM). When compared to kirenol (IC_50_ = 1.73 ± 0.3 μM), the majority of the compounds exhibited improved anti-inflammatory action against RAW 264.7 mouse monocyte–macrophage [62]. This suggests that altering the structure of kirenol could effectively enhance its anti-inflammatory properties. Wang et al. [63] discovered two 14,16-epoxy-pimarane diterpenoids (**31** and **32**) and five 8,15-epoxy-pimarane diterpenoids (**33–37**) from kirenol, and showed moderate to weak inhibitory effects on factor Xa (FXa) [63]. Furthermore, Wang et al. [64] also reported the semi-synthesis of strobol C (**38**), an ent-strobane derived from kirenol via a Wagner–Meerwein rearrangement with inhibitory potential for FXa (IC_50_ = 81.22 nM). Aside from that, combination therapy is an effective way to boost kirenol′s pharmacokinetics and anti-inflammatory effect and can be explored further in the future. Figure 7 depict the possible structural modifications and derivatives of kirenol [62,63,64].

## 6. Conclusions and Future Perspectives

**Figure 8:**
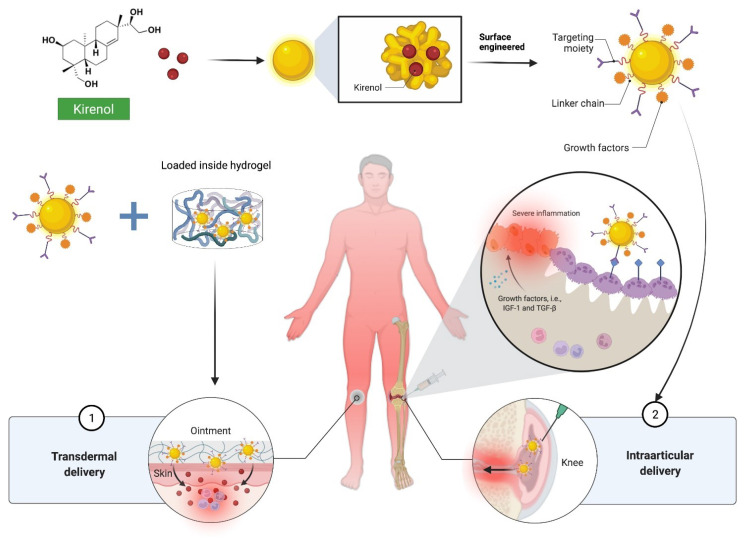
Future prospects for a dual anti-inflammatory strategy involving kirenol-loaded gold nanoparticles. To promote internalisation into the targeted location, the surface of the nanoparticles can be decorated with targeting ligands such as cell-penetrating peptides and monoclonal antibody tocilizumab, which is an interleukin-6 (IL-6) blocker. The recommended technique, when coupled, may have a synergistic effect in lowering inflammation. Abbreviations: IGF-1, insulin-like growth factor 1; TGF- β, transforming growth factor beta.

Kirenol possesses potent anti-inflammatory properties with (1) the suppression of pro-inflammatory cytokines, reduction in NF-κB, (2) the attenuation of antioxidant enzymes and activation of HO-1 expression, and (3) Nrf2 phosphorylation among the main molecular mechanisms contributing to its anti-inflammatory effects. Kirenol is effective in the treatment of inflammation-related multiple sclerosis, inflammatory bowel disease, diabetic wounds, arthritis, cardiovascular disease, and other joint diseases. Nevertheless, kirenol′s therapeutic value is limited by its lack of physicochemical and pharmacokinetic features. Researchers are now looking into the advantages of combining kirenol with other compounds and modifying its chemical structure to improve its systemic bioavailability. Overall, kirenol′s anti-inflammatory properties have been extensively elucidated by a significant body of research, which can serve as a foundation in the development and clinical use of potent drugs with good therapeutic potentials (Figure 8). Based on the scientific evidence presented in this review, we believe that kirenol is a promising natural lead molecule for a new drug design, development, and therapy for inflammation. However, more preclinical research, pharmacokinetics, and bioavailability studies are needed to confirm and strengthen this molecule into the next stage of drug discovery and development for the treatment of inflammation.

## Figures and Tables

**Table 1:** Docked study results of kirenol with the inflammation target proteins.

S. No	Protein	Name of the Protein	Ligand	MolDock Score	Rerank Score	HBond	Amino Acid Residue ID
1.	5IKR	COX-2	Kirenol	−91.509	−78.647	−4.718	5IKR (B): Arg 120, Glu 524, Leu 80, Leu 123, Leu 472, Lys 83, Met 471, Phe 470, Pro 84, Pro 86, Ser 119, Thr 85, Tyr 115, HOH 754 (B) (water) 107.
2.	6NCF	LOX-5	Kirenol	−82.439	−72.048	−9.983	6NCF (B): Arg 47, Arg 401, Asn 613, Asp 170, Gln 15, Glu 16, Glu 612, Glu 614, Glu 622, Leu 615, Phe 402, Ser 14.
3.	4RWS	CXCR4	Kirenol	−69.913	−59.367	−9.542	4RWS (A): Arg 188, Asp 262, Cys 187, His 113, Ile 259, Ile 284, Leu 266, Tyr 190, Tyr 255, 4RWS (C): Ala 3, Cys 5, Gly 2, His 6, Leu 1.
4.	6AK3	Human prostaglandin E receptor EP3	Kirenol	−83.287	−66.223	−1.442	6AK3 (A): Gly 65, Ile 340, Leu 60, Leu 341, Leu 349, Thr 61, Trp 344, Val 64, 6AK3 (B): Gly 65, Leu 60, Leu 341, Thr 61, Trp 344, Val 64.

**Table 2:** Physicochemical and drug-likeness properties of kirenol.

Property/Rule	Result
Molecular formula	C_20_H_34_O_4_
Molecular weight	338.50
Hydrogen bond donors	4
Hydrogen bond acceptors	4
Rotatable bonds	3
*Log P* (Partition coefficient, Predicted value)	1.898
Molar refractivity	95.87 cm^3^
Topological polar surface area	80.9 Å²
Lipinski’s Rule of Five	Passed
Ghose_Filter	Passed
Veber’s Rule	Passed
BBB Likeness Rule	Passed
Unweighted QED	Passed
Weighted QED	Passed

Abbreviations: BBB, blood–brain barrier; QED, quantitative estimate of drug-likeness.

## Data Availability

The data presented in this study are available on request from the corresponding author.

## References

[1] Dinarello C.A. (2010). Anti-inflammatory agents: Present and future. Cell.

[2] Mack M. (2018). Inflammation and fibrosis. Matrix Biol..

[3] Mittal M., Siddiqui M.R., Tran K., Reddy S.P., Malik A.B. (2014). Reactive oxygen species in inflammation and tissue injury. Antioxid. Redox Signal..

[4] Brady J., Horie S., Laffey J.G. (2020). Role of the adaptive immune response in sepsis. Intensive Care Med. Exp..

[5] Lee H., Fessler M.B., Qu P., Heymann J., Kopp J.B. (2020). Macrophage polarization in innate immune responses contributing to pathogenesis of chronic kidney disease. BMC Nephrol..

[6] Lugrin J., Rosenblatt-Velin N., Parapanov R., Liaudet L. (2014). The role of oxidative stress during inflammatory processes. Biol. Chem..

[7] Medzhitov R. (2008). Origin and physiological roles of inflammation. Nature.

[8] Iwasaki A., Medzhitov R. (2015). Control of adaptive immunity by the innate immune system. Nat. Immunol..

[9] Nathan C. (2002). Points of control in inflammation. Nature.

[10] Fitzgerald K.A., Kagan J.C. (2020). Toll-like receptors and the control of immunity. Cell.

[11] Liu J., Cao X. (2016). Cellular and molecular regulation of innate inflammatory responses. Cell. Mol. Immunol..

[12] Chen L., Deng H., Cui H., Fang J., Zuo Z., Deng J., Li Y., Wang X., Zhao L. (2018). Inflammatory responses and inflammation-associated diseases in organs. Oncotarget.

[13] Koeberle A., Werz O. (2014). Multi-target approach for natural products in inflammation. Drug Discov. Today.

[14] Ci X., Li H., Yu Q., Zhang X., Yu L., Chen N., Song Y., Deng X. (2009). Avermectin exerts anti-inflammatory effect by downregulating the nuclear transcription factor kappa-B and mitogen-activated protein kinase activation pathway. Fundam. Clin. Pharmacol..

[15] French J.A., Koepp M., Naegelin Y., Vigevano F., Auvin S., Rho J.M., Rosenberg E., Devinsky O., Olofsson P.S., Dichter M.A. (2017). Clinical studies and anti-inflammatory mechanisms of treatments. Epilepsia.

[16] Ponder A., Long M.D. (2013). A clinical review of recent findings in the epidemiology of inflammatory bowel disease. Clin. Epidemiol..

[17] Furman D., Campisi J., Verdin E., Carrera-Bastos P., Targ S., Franceschi C., Ferrucci L., Gilroy D.W., Fasano A., Miller G.W. (2019). Chronic inflammation in the etiology of disease across the life span. Nat. Med..

[18] Calixto J.B., Otuki M.F., Santos A.R. (2003). Anti-inflammatory compounds of plant origin. Part I. Action on arachidonic acid pathway, nitric oxide and nuclear factor κ B (NF-κB). Planta Med..

[19] Zhu F., Du B., Xu B. (2018). Anti-inflammatory effects of phytochemicals from fruits, vegetables, and food legumes: A review. Crit. Rev. Food Sci. Nutr..

[20] Li H., Li P.Y., Yeung P. (2013). A simple HPLC assay for ginsenoside-Rh2 in plasma and its application for pharmacokinetic study in rats. Nat. Prod. Chem. Res..

[21] Ibrahim S.R., Altyar A.E., Sindi I.A., El-Agamy D.S., Abdallah H.M., Mohamed S.G., Mohamed G.A. (2021). Kirenol: A promising bioactive metabolite from siegesbeckia species: A detailed review. J. Ethnopharmacol..

[22] Stils Jr H.F. (2005). Adjuvants and antibody production: Dispelling the myths associated with Freund’s complete and other adjuvants. ILAR J..

[23] Billiau A., Matthys P. (2001). Modes of action of Freund’s adjuvants in experimental models of autoimmune diseases. J. Leukoc. Biol..

[24] Wang J.-P., Zhou Y.-M., Ye Y.-J., Shang X.-M., Cai Y.-L., Xiong C.-M., Wu Y.-X., Xu H.-X. (2011). Topical anti-inflammatory and analgesic activity of kirenol isolated from Siegesbeckia orientalis. J. Ethnopharmacol..

[25] Ransohoff R.M. (2016). How neuroinflammation contributes to neurodegeneration. Science.

[26] Xiao J., Yang R., Yang L., Fan X., Liu W., Deng W. (2015). Kirenol attenuates experimental autoimmune encephalomyelitis by inhibiting differentiation of Th1 and th17 cells and inducing apoptosis of effector T cells. Sci. Rep..

[27] Lee M., Kim S.H., Lee H.K., Cho Y., Kang J., Sung S.H. (2014). ent-kaurane and ent-pimarane diterpenes from Siegesbeckia pubescens inhibit lipopolysaccharide-induced nitric oxide production in BV2 microglia. Biol. Pharm. Bull..

[28] Rajendran P., Alzahrani A.M., Ahmed E.A., Veeraraghavan V.P. (2021). Kirenol inhibits B[a]P-induced oxidative stress and apoptosis in endothelial cells via modulation of the Nrf2 signaling pathway. Oxidative Med. Cell. Longev..

[29] Lin F.C.-F., Lee S.-S., Li Y.-C., Ho Y.-C., Chen W.-Y., Chen C.-J., Lee M.-W., Yeh K.-L., Tsai S.C.-S., Kuan Y.-H. (2021). Protective Effects of Kirenol against Lipopolysaccharide-Induced Acute Lung Injury through the Modulation of the Proinflammatory NFκB Pathway and the AMPK2-/Nrf2-Mediated HO-1/AOE Pathway. Antioxidants.

[30] Baumgart D.C., Carding S.R. (2007). Inflammatory bowel disease: Cause and immunobiology. Lancet.

[31] Liu X.H., Du Y.J., Liu G.X., Dan G.M., Tong X., Xiao J. (2019). Kirenol relieves dextran sulfate sodium-induced ulcerative colitis in mice by inhibiting inflammatory cytokines and inducing CD4^+^ T lymphocyte apoptosis. J. South. Med. Univ..

[32] Gupta A., Upadhyay N.K., Sawhney R., Kumar R. (2008). A poly-herbal formulation accelerates normal and impaired diabetic wound healing. Wound Repair Regen..

[33] Spampinato S.F., Caruso G.I., De Pasquale R., Sortino M.A., Merlo S. (2020). The treatment of impaired wound healing in diabetes: Looking among old drugs. Pharmaceuticals.

[34] Burgess J.L., Wyant W.A., Abdo Abujamra B., Kirsner R.S., Jozic I. (2021). Diabetic Wound-Healing Science. Medicina.

[35] Ren J., Yang M., Chen J., Ma S., Wang N. (2020). Anti-inflammatory and wound healing potential of kirenol in diabetic rats through the suppression of inflammatory markers and matrix metalloproteinase expressions. Biomed. Pharmacother..

[36] Lewiecki E.M. (2011). New targets for intervention in the treatment of postmenopausal osteoporosis. Nat. Rev. Rheumatol..

[37] Niu Y., Li Y., Kong X., Zhang R., Sun Y., Li Q., Li C., Liu L., Wang J., Mei Q. (2012). The beneficial effect of Radix Dipsaci total saponins on bone metabolism in vitro and in vivo and the possible mechanisms of action. Osteoporos. Int..

[38] Zou B., Zheng J., Deng W., Tan Y., Jie L., Qu Y., Yang Q., Ke M., Ding Z., Chen Y. (2021). Kirenol inhibits RANKL-induced osteoclastogenesis and prevents ovariectomized-induced osteoporosis via suppressing the Ca2+-NFATc1 and Cav-1 signaling pathways. Phytomedicine.

[39] Kim M.-B., Song Y., Hwang J.-K. (2014). Kirenol stimulates osteoblast differentiation through activation of the BMP and Wnt/β-catenin signaling pathways in MC3T3-E1 cells. Fitoterapia.

[40] Karaman İ., Günay A.E., Yerer M.B., Demirpolat E., Doğan S., Yay A.H., Kafadar İ.H. (2020). Effect of kirenol on the interaction between the WNT/β-Catenin and RUNX2/TCF/LEF1 pathways in fracture healing in vivo. Acta Orthop. Et Traumatol. Turc..

[41] Wu J., Li Q., Jin L., Qu Y., Liang B.-B., Zhu X.-T., Du H.-Y., Jie L.-G., Yu Q.-H. (2019). Kirenol inhibits the function and inflammation of fibroblast-like synoviocytes in rheumatoid arthritis in vitro and in vivo. Front. Immunol..

[42] Wang Z.-M., Zhu S.-G., Wu Z.-W., Lu Y., Fu H.-Z., Qian R.-Q. (2011). Kirenol upregulates nuclear Annexin-1 which interacts with NF-κB to attenuate synovial inflammation of collagen-induced arthritis in rats. J. Ethnopharmacol..

[43] Lu Y., Xiao J., Wu Z., Wang Z., Fu H., Chen Y., Qian R. (2012). Effects of kirenol on bovine type II collagen-induced rat lymphocytes in vivo and in vitro. Nan Fang Yi Ke Da Xue Xue Bao J. South. Med. Univ..

[44] Lu Y., Xiao J., Wu Z.-W., Wang Z.-M., Hu J., Fu H.-Z., Chen Y.-Y., Qian R.-Q. (2012). Kirenol exerts a potent anti-arthritic effect in collagen-induced arthritis by modifying the T cells balance. Phytomedicine.

[45] Wu J., Qu Y., Deng J.-X., Liang W.-Y., Jiang Z.-L., Lai R., Yu Q.-H. (2017). Molecular docking studies of kirenol a traditional Chinese medicinal compound against rheumatoid arthritis cytokine drug targets (TNF-α, IL-1 and IL-6). Biomed. Res..

[46] Mogensen T.H. (2009). Pathogen recognition and inflammatory signaling in innate immune defenses. Clin. Microbiol. Rev..

[47] Kany S., Vollrath J.T., Relja B. (2019). Cytokines in inflammatory disease. Int. J. Mol. Sci..

[48] Hayden M., West A., Ghosh S. (2006). NF-κ B and the immune response. Oncogene.

[49] Cross M.L., Ganner A., Teilab D., Fray L.M. (2004). Patterns of cytokine induction by gram-positive and gram-negative probiotic bacteria. FEMS Immunol. Med. Microbiol..

[50] Prescott J.A., Mitchell J.P., Cook S.J. (2021). Inhibitory feedback control of NF-κB signalling in health and disease. Biochem. J..

[51] Bogdan C. (2001). Nitric oxide and the immune response. Nat. Immunol..

[52] Orlando B.J., Malkowski M.G. (2016). Substrate-selective inhibition of cyclooxygeanse-2 by fenamic acid derivatives is dependent on peroxide tone. J. Biol. Chem..

[53] Gilbert N.C., Gerstmeier J., Schexnaydre E.E., Börner F., Garscha U., Neau D.B., Werz O., Newcomer M.E. (2020). Structural and mechanistic insights into 5-lipoxygenase inhibition by natural products. Nat. Chem. Biol..

[54] Berlinck R.G., Bernardi D.I., Fill T., Fernandes A.A., Jurberg I.D. (2021). The chemistry and biology of guanidine secondary metabolites. Nat. Prod. Rep..

[55] Kircher M., Herhaus P., Schottelius M., Buck A.K., Werner R.A., Wester H.-J., Keller U., Lapa C. (2018). CXCR4-directed theranostics in oncology and inflammation. Ann. Nucl. Med..

[56] Morimoto K., Suno R., Hotta Y., Yamashita K., Hirata K., Yamamoto M., Narumiya S., Iwata S., Kobayashi T. (2019). Crystal structure of the endogenous agonist-bound prostanoid receptor EP3. Nat. Chem. Biol..

[57] Song X.L., Zhang Q.Y., Wang Z.M., Fu H.Z., Qian R.Q. (2011). A rapid and simple RP-HPLC method for quantification of kirenol in rat plasma after oral administration and its application to pharmacokinetic study. Biomed. Chromatogr..

[58] Huo L., Jiang Z., Lei M., Wang X., Guo X. (2013). Simultaneous quantification of Kirenol and ent-16β, 17-dihydroxy-kauran-19-oic acid from *Herba siegesbeckiae* in rat plasma by liquid chromatography–tandem mass spectrometry and its application to pharmacokinetic studies. J. Chromatogr. B.

[59] Yin Y., Sun Y., Jiang Z. (2016). Pharmacokinetics study of two active diterpenoids from *Herba siegesbeckiae* in rat plasma. Yao Xue Xue Bao = Acta Pharm. Sin..

[60] Martinho N., Damgé C., Reis C.P. (2011). Recent advances in drug delivery systems. J. Biomater. Nanobiotechnol..

[61] Jahangirian H., Lemraski E.G., Webster T.J., Rafiee-Moghaddam R., Abdollahi Y. (2017). A review of drug delivery systems based on nanotechnology and green chemistry: Green nanomedicine. Int. J. Nanomed..

[62] Wang J.L., Sun H.Y., Li Y.F., Chu H.P., Sun J.Y. (2020). Synthesis and preliminary anti-inflammatory activity exploration of novel derivatives of kirenol. N. J. Chem..

[63] Wang J., Wu M., Gao C., Fu H. (2019). Semisynthesis of epoxy-pimarane diterpenoids from kirenol and their FXa inhibition activities. Bioorganic Med. Chem..

[64] Wang J., Ma H., Fu H. (2018). Semisynthesis of ent-norstrobane diterpenoids as potential inhibitor for factor Xa. Bioorganic Med. Chem. Lett..

